# Discoidin domain receptor 1 modulates insulin receptor signaling and biological responses in breast cancer cells

**DOI:** 10.18632/oncotarget.18020

**Published:** 2017-05-19

**Authors:** Veronica Vella, Roberta Malaguarnera, Maria Luisa Nicolosi, Chiara Palladino, Cristina Spoleti, Michele Massimino, Paolo Vigneri, Michele Purrello, Marco Ragusa, Andrea Morrione, Antonino Belfiore

**Affiliations:** ^1^ School of Motor Sciences, Faculty of Human and Social Sciences, Kore University of Enna, Enna, Italy; ^2^ Endocrinology, Department of Health Sciences, University Magna Graecia of Catanzaro, Catanzaro, Italy; ^3^ Department of Clinical and Experimental Medicine, Faculty of Medicine, University of Catania, Catania, Italy; ^4^ Department of Biomedical and Biotechnological Sciences, Unit of BioMolecular, Genome, and Complex System BioMedicine, University of Catania, Catania, Italy; ^5^ Department of Urology and Biology of Prostate Cancer Program, Sidney Kimmel Cancer Center, Thomas Jefferson University, Philadelphia, PA, USA

**Keywords:** insulin receptor, insulin receptor isoforms, DDR1, breast cancer

## Abstract

The fetal isoform A of the insulin receptor (IR-A) is frequently overexpressed in a variety of malignancies including breast cancer. IR overexpression has a recognized role in cancer progression and resistance to anticancer therapies. In particular, IR-A has a peculiar mitogenic potential and is activated not only by insulin but also by IGF-2. Previously, we identified discoidin domain receptor 1 (DDR1) as a new IR-A interacting protein. DDR1, a non-integrin collagen tyrosine kinase receptor, is overexpressed in several malignancies and plays a role in cancer progression and metastasis.

We now evaluated whether DDR1 is able to exert a role in breast cancer biology by functionally cross-talking with IR. In MCF-7 human breast cancer cells, IR and DDR1 co-immunoprecipitated and co-localized after insulin or IGF-2 stimulation. In a panel of breast cancer cells, DDR1 knockdown by specific siRNAs markedly inhibited IR downstream signaling as well as proliferation, migration and colony formation in response to insulin and IGF-2. These effects were accompanied by reduction of IR protein and mRNA expression, which involved both transcriptional and post-transcriptional effects. DDR1 overexpression elicited opposite effects. Bioinformatics analysis of public domain databases showed that IR and DDR1 co-expression significantly correlates with several clinically relevant histopathological and molecular features of human breast carcinomas.

These findings demonstrate that, in human breast cancer cells, DDR1 regulates IR expression and ligand dependent biological actions. This novel functional crosstalk is likely clinically relevant and may become a new molecular target in breast cancer.

## INTRODUCTION

Dysregulation of the insulin/IGF signaling (IIGFs), involving the overexpression of receptors for IGF-1 and/or insulin (IGF-1R and IR) and/or cognate ligands (IGF-1, IGF-2), has an important role in the early phases of carcinogenesis of breast cancer, and is associated with cancer progression and metastases and resistance to a variety of therapies [1–4]. IIGFs is especially relevant to Epithelial Mesenchymal Transition (EMT) [5] and other stem-like features [6], which play a key role in cancer development and recurrence. However, IGF-1R inhibitors have shown limited benefit in cancer when used as single therapy [7–10]. Thus, to exploit the anticancer potential of IIGFs inhibition, there is an urgent need to better understand IIGFs activities and interactions. A deep understanding of the molecular mechanisms for this failure will open the way to successful therapies able to eradicate breast cancer. A major mechanism of resistance to anti-IGF-1R drugs involves the IR, which is commonly overexpressed in breast cancer and predominantly expressed as the so called ‘fetal isoform’ (IR-A), which is a *bona fide* receptor for IGF-2 and proinsulin [11, 12]. Significantly, the IR-A/IGF-2 autocrine loop plays a key role in many cancer histotypes, including breast cancer [13, 14].

Notably, IRs and IGF-1R signaling and actions may undergo diversification following crosstalk with other membrane receptors. In a previous study, aimed at discovering new substrates/mediators of the IGF-2/IR-A pathway, we reported that DDR1 is found in multiprotein complexes associated with tyrosine-phosphorylated IR in response to IGF-2 and to a lesser extent to insulin [15]. DDR1 belongs to the discoidin domain receptors (DDRs), family, which includes two members, DDR1 and DDR2 recognized as collagen receptors [16, 17]. Upon binding to collagens, DDR1 undergoes slow but prolonged phosphorylation at several tyrosine residues, which potentially serve as binding sites of Src-homology-2 (SH2) and phosphotyrosine binding (PTB) domain-containing molecules [18]. DDR1 is often overexpressed in cancer, and plays a critical role in cancer cell migration, EMT, and metastases [19–22]. We recently reported that, in breast cancer cells, IGF-1R functionally crosstalks with DDR1 and this interaction increases IGF-1R stability and enhances protumorigenic actions of IGF-1 [23]. In turn, IGF-1, as well as IGF-2 and insulin, induce DDR1 upregulation and activation establishing a positive feedback of IIGFs [24].

In the present work we aimed at elucidating the biological role of the IR - DDR1 crosstalk in human breast cancer cells. These results indicate that this functional interaction plays a significant role in human breast cancer and may become a viable target for therapy.

## RESULTS

### DDR1 and IR expression in breast cancer cells

**Figure 1:**
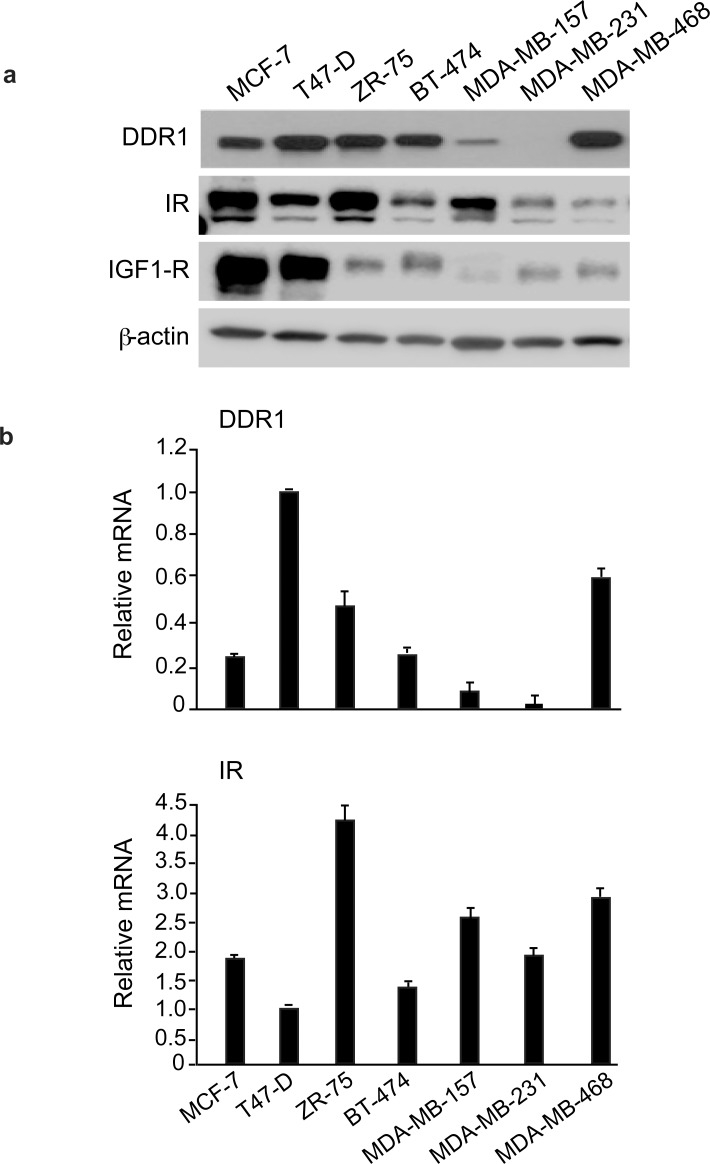
IR, DDR1 and IGF-1R expression in cultured cells **(a)** DDR1, IR and IGF-1R protein expression in various cell lines. A panel of human breast cancer cell lines (MCF-7, T47-D, ZR-75, BT-474, MDA-MB-157, MDA-MB-231, MDA-MB-468) were analyzed by western immunoblot for DDR1, IR and IGF-1R expression using specific polyclonal antibodies, as indicated. β-actin antibody was used as control for protein loading. A representative blot of three independent experiments is shown. **(b)** qRT-PCR analysis of DDR1 and IR mRNA. Human DDR1 and IR mRNA levels were evaluated in all human cell lines shown in panel **(a)**. Normalization was done using human β-actin as housekeeping control gene. Data are presented as the mean±SEM (error bars) from three independent experiments.

We first evaluated by immunoblot the expression of DDR1 and IR in a panel of human breast cancer cell lines (MCF-7, T47D, ZR-75, BT-474, MDA-MB-157, MDA-MB-231, MDA-MB-468). Both molecules were expressed at variable levels with the highest DDR1 levels observed in MCF-7, T47D, ZR-75, BT-474 and MDA-MB-468, and the highest IR levels observed in MCF-7, ZR-75 and MDA-MB-157 cells (Figure 1a). DDR1 and IR mRNA expression, measured by quantitative real-time RT-PCR (qRT–PCR), was generally in good agreement with the immunoblot results (Figure 1b).

MCF-7, BT-474 and MDA-MB-157 breast cancer cells were chosen for subsequent experiments. All these cells have ductal characteristics and metastatic potential, and all respond to insulin [25]. MCF-7 and BT-474 are estrogen receptor positive, while MDA-MB-157 cells have characteristics of triple negative cells. BT-474 cells are also HER-2 positive and tamoxifen-resistant. Both MCF-7 and BT-474 cells expressed high levels of DDR1 and IR, with MCF-7 expressing higher IR levels than BT-474. MDA-MB-157 showed low levels of DDR1 and intermediate levels of IR. The IGF-1R was expressed at high levels in MCF-7 cells, low levels in MDA-MB-157 and intermediate levels in BT-474 cells (Figure 1a).

### DDR1 and IR interact in breast cancer cells

**Figure 2:**
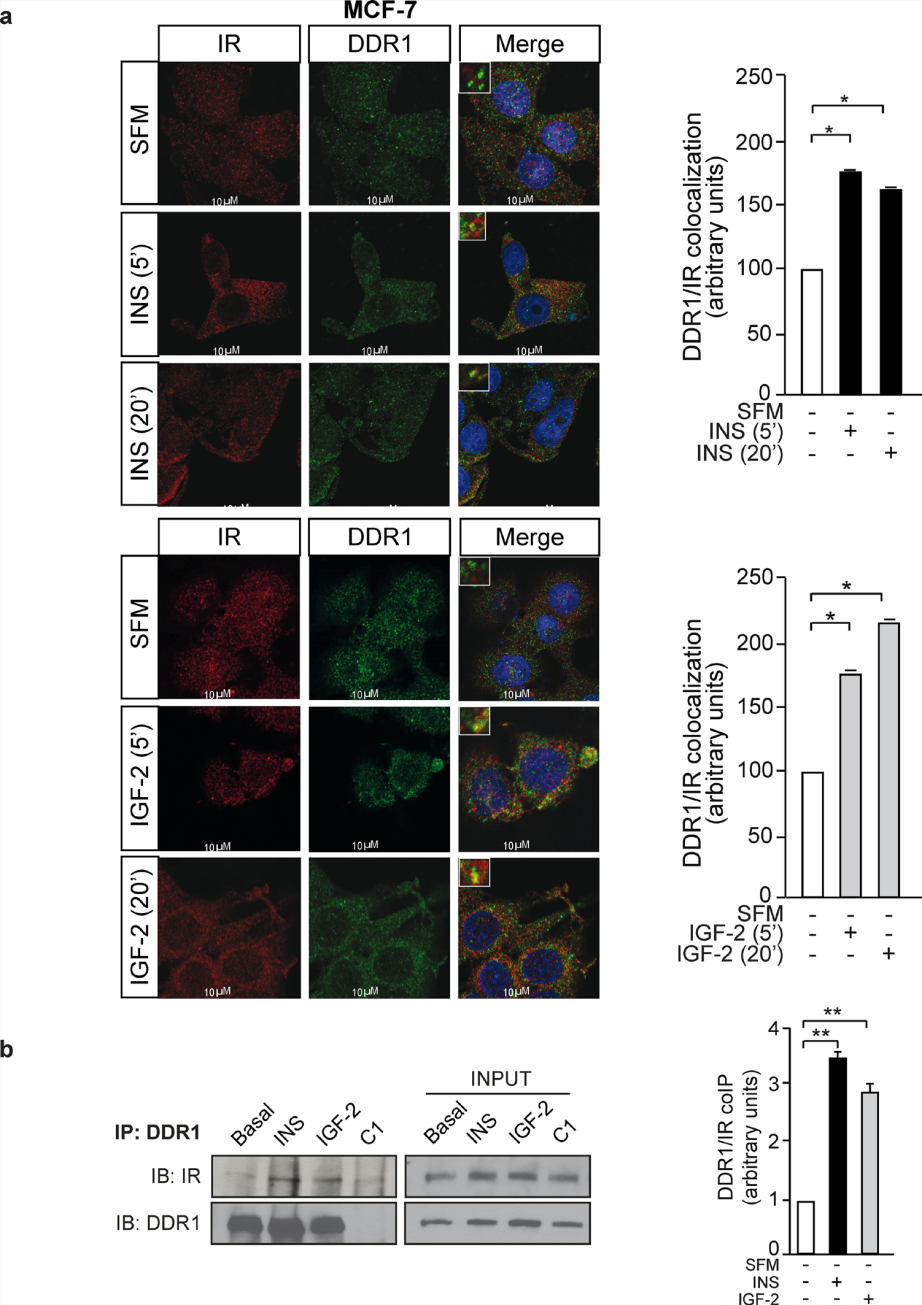
IR and DDR1 co-localization **(a)** IR and DDR1 co-localize in breast cancer cells. MCF-7 cells were plated onto coverslips and serum-starved for 6h. Cells were then stimulated with 10nM of either insulin (upper panel) or IGF-2 (lower panel) for the indicated times. The staining indicating co-localization of the IR with DDR1 was assessed by confocal microscopy. Graphs on the right showing the co-localization index, calculated by ImageJ software, represent the mean±SEM of two independent experiments. **(b)** IR and DDR1 co-immunoprecipitate in MCF-7 breast cancer cells. Insulin and IGF-2 stimulation increases IR co-immunoprecipitation with DDR1. Cells were serum starved for 24h and stimulated with either insulin or IGF-2 (10nM) for 5min. Cells were then solubilized and total lysates were (C1). Immunoprecipitated with anti-DDR1 specific antibody. Negative control, including the use of an unrelated primary antibody, is also shown. An aliquot of each fraction (input) was evaluated as control. Filters were probed with anti-DDR1 or anti-IR antibodies, as indicated. A representative blot of three independent experiments is shown. The graph on the right represents the mean±SEM of the densitometric analysis where IR signal was normalized against total DDR1. **(a-b)** *0.01 < p < 0.05; **0.001 < p < 0.01; (basal *vs*. insulin or IGF-2). Statistical significance was calculated using Student's t-test.

In order to evaluate whether the DDR1 and IR co-localize in breast cancer cells, MCF-7 cells were plated onto coverslips, serum-starved for 24h and stimulated with either insulin or IGF-2 at a dose of 10nM for 5 and 20 min. Cells were then stained with anti-DDR1 and anti-IR antibodies and examined by confocal microscopy. In unstimulated cells, IR and DDR1 were mainly expressed at the plasma membrane with minimal co-localization observed. After cell stimulation with either insulin (Figure 2a, upper panel) or IGF-2 (Figure 2a, lower panel), both IR and DDR1 were rapidly internalized and co-localized in both the cytoplasm and perinuclear compartments. These results were confirmed by co-immunoprecipitation. MCF-7 cells were serum starved, treated or not with either insulin or IGF-2 (10nM for 5min), lysed and immunoprecipitated with anti-DDR1 antibody. Immunoprecipitates were then blotted with either anti-DDR1 or anti-IR antibodies, respectively (Figure 2b). DDR1 co-precipitated with the IR and this interaction was enhanced after insulin and IGF-2 stimulation (Figure 2b).

### DDR1 expression levels regulate insulin and IGF-2 biological effects

**Figure 3:**
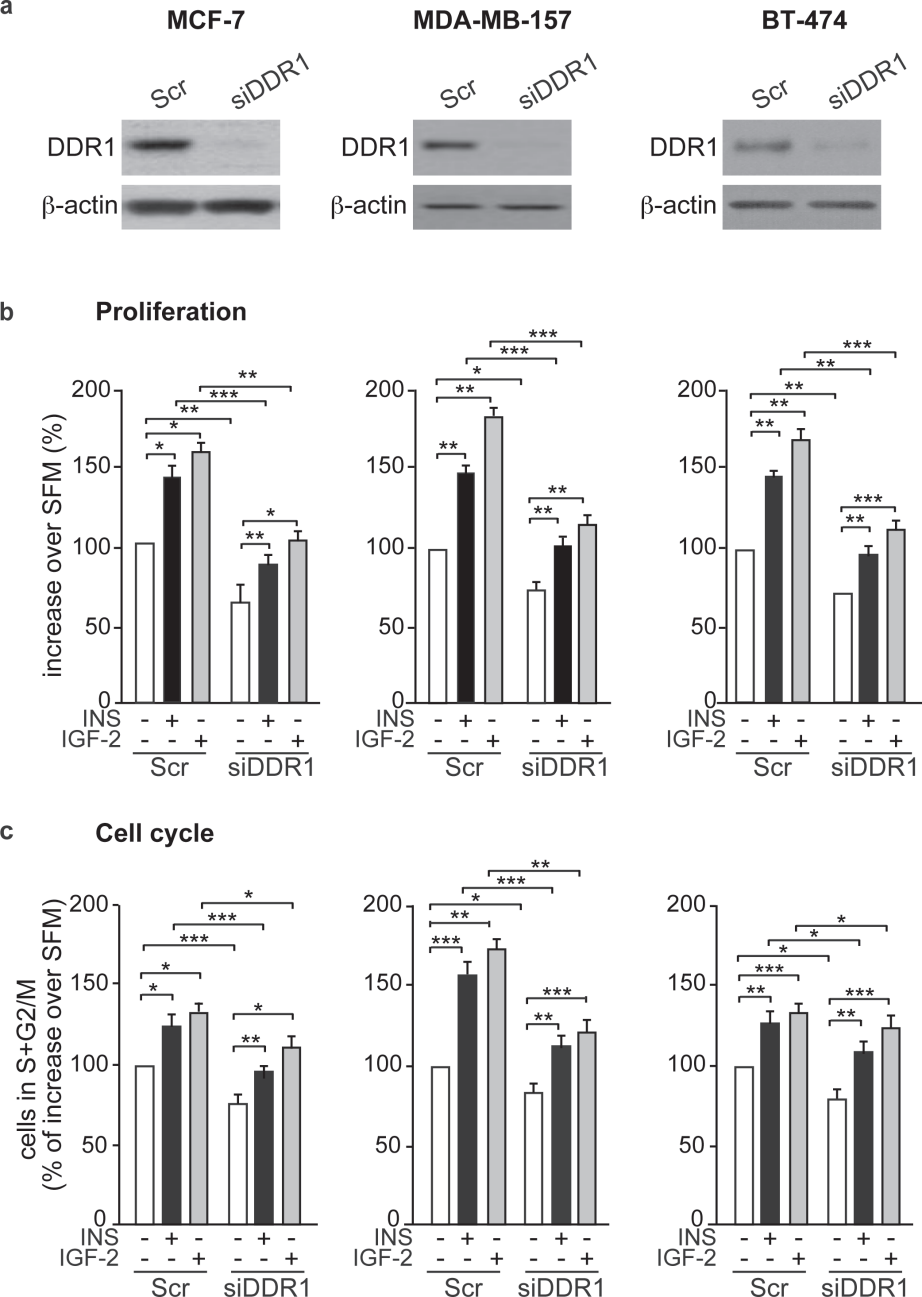

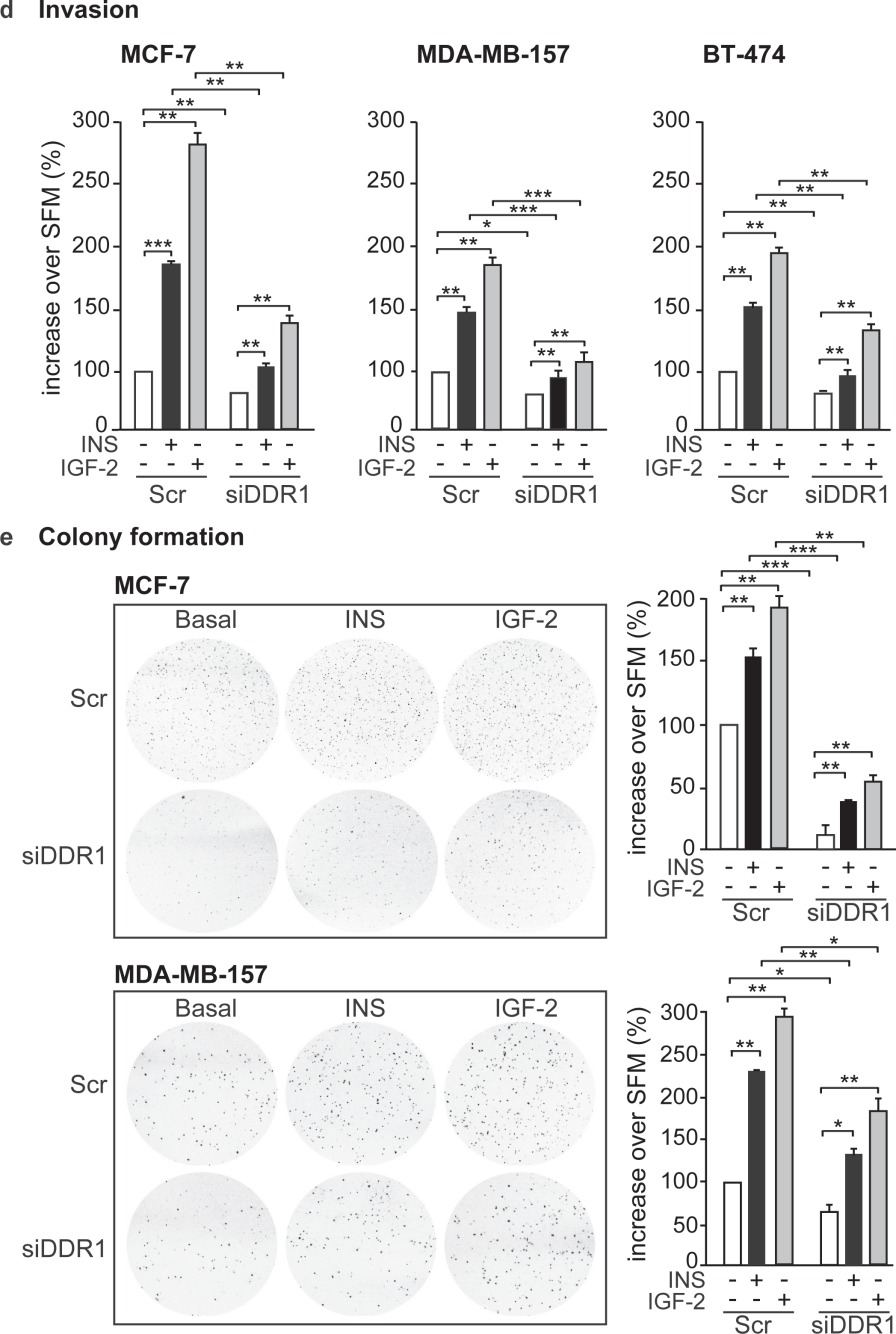
DDR1 depletion affects insulin and IGF-2 mediated biological effects in human cancer cells **(a)** Western blot before and after DDR1 depletion. MCF-7, MDA-MB-157 and BT-474 breast cancer cells were transiently transfected with either a pool of four DDR1 siRNA oligos or scramble siRNA oligos. After 24h, cells were grown in medium containing 2.5% of CS-FCS for 24h. DDR1 depletion was confirmed for each cells line by western blot analysis as shown in the panel. **(b)** Cell proliferation. Cell viability was evaluated by MTT assay. Values are expressed as percentages of untreated scramble-transfected cells (basal) and represent the mean±SEM of three independent experiments in triplicate. **(c)** Cell cycle progression. MCF-7, MDA-MB-157 and BT-474 breast cancer cells were transiently transfected as in **(a)**. After 24h, cells were grown in medium containing 0.1% of BSA for additional 24h. Cells were then incubated with or without insulin or IGF-2 at a dose of 10nM for additional 48h and analyzed for cell-cycle profiles. Cell populations positive for propidium iodine staining were evaluated by FACS analysis, and G0/G1 and G2/M phases were scored. The graph shows the percentage of cells in S and G2/M phases. Values are expressed as percent of basal (untreated scramble transfected cells) and are the mean±SEM of three independent experiments. **(d)** Cell invasion. MCF-7, MDA-MB-157 and BT-474 breast cancer cells were transiently transfected as in **(a)**. After 24h, cells were grown in medium containing 0.1% of BSA for additional 24 h. Cells were then removed from plates with 0.01% trypsin and seeded on polycarbonate filters coated with 25μg/mL fibronectin. Cells were allowed to migrate for 6h (MCF-7 and MDA-MB-157) or 8h (BT-474 cells) in response to 10nM of insulin or IGF-2 added to the lower chamber. Values are mean±SEM of three independent experiments done in duplicate and are expressed as percent increase over untreated scramble cells (basal). **(e)** Colony formation. MCF-7, MDA-MB-157 and BT-474 breast cancer cells were transiently transfected as in **(a)**, and seeded in soft-agar, as described in Materials and Methods. Cells were plated in triplicate and cultured in serum free medium containing 2.5% CS-FCS for 3 weeks. Colonies developed only from plated MCF-7 and MDA-MB-157 cells but not from BT-474. Colonies were stained with MTT and then photographed. The histogram represents the mean number of colonies shown in **(e)**. Error bars indicate SEM (n = 3 wells). **(b-e)** *0.01 < p < 0.05; **0.001 < p < 0.01; ***p < 0.001. Statistical significance was calculated using one-way ANOVA followed by Bonferroni test.

**Figure 4:**
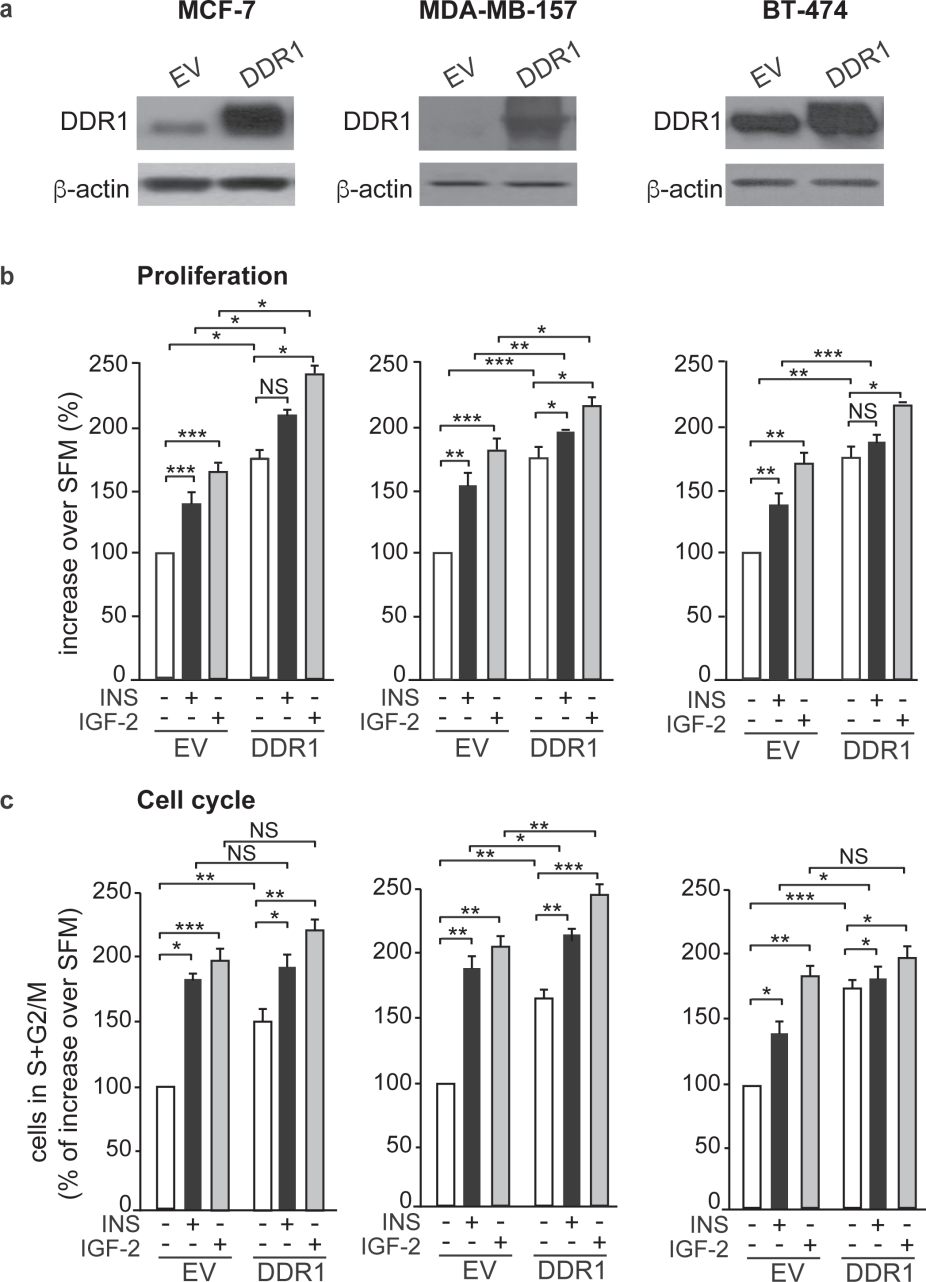

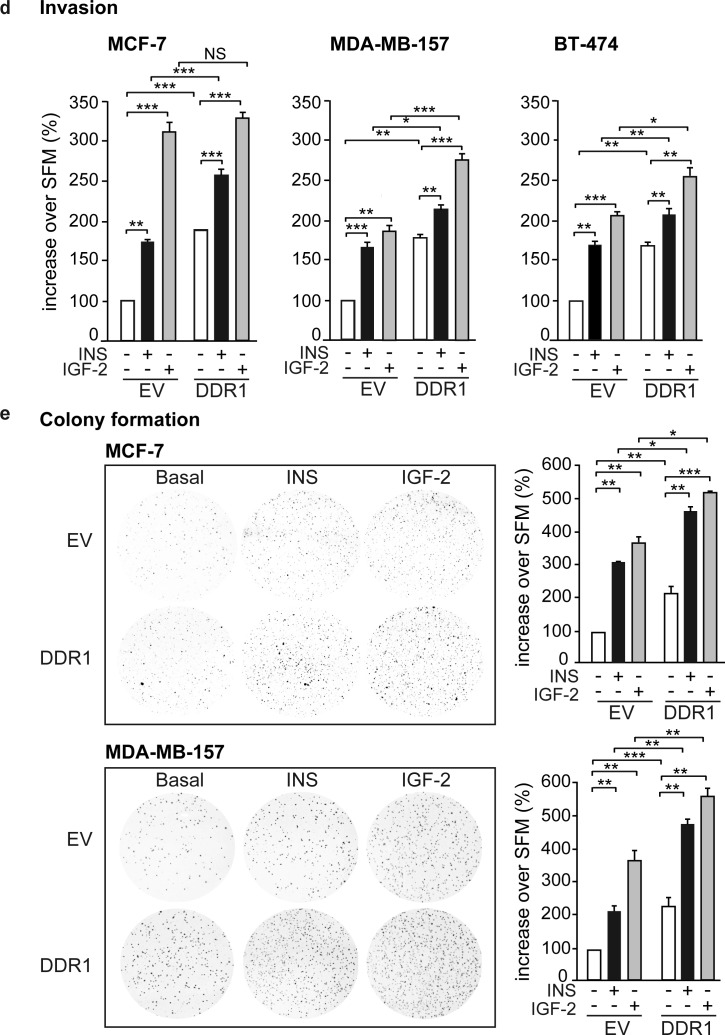
DDR1 overexpression affects insulin and IGF-2 mediated biological effects in human cancer cells **(a)** Western blot before and after DDR1 overexpression. MCF-7, MDA-MB-157 and BT-474 breast cancer cells were transiently transfected with either constitutive empty (pCMV6-EV) or human DDR1 (pCMV6-DDR1) expressing vectors. After 24h, cells were grown in medium containing 2.5% of CS-FCS for 24h and then stimulated with 10nM of insulin or IGF-2. DDR1 overexpression was confirmed for each cells line by western blot analysis. **(b)** cell proliferation. Breast cancer cells were transfected as in **(a)**. After 24h, cells were grown in medium containing 0.1% of BSA for additional 24h and then stimulated with 10nM of insulin or IGF-2 for 48h. Cell viability was evaluated by MTT assay. Values are expressed as percentages of empty vector-transfected cells (basal) and represent the mean±SEM of three independent experiments in triplicate. **(c)** Cell cycle progression. MCF-7, MDA-MB-157 and BT-474 breast cancer cells were transiently transfected as in **(a)**. After 24h, cells were grown in medium containing 0.1% of BSA for additional 24h. Cells were then incubated with or without insulin or IGF-2 at a dose of 10nM for additional 48h and analyzed for cell-cycle profiles. Cell populations positive for propidium iodine staining were evaluated by FACS analysis, and G0/G1 and G2/M phases were scored. The graph shows the percentage of cells in S and G2/M phases. Values are expressed as percent of basal (untreated scramble transfected cells) and are the mean±SEM of three independent experiments. **(d)** Cell invasion. MCF-7, MDA-MB-157 and BT-474 breast cancer cells were transiently transfected as in **(a)**. After 24h, cells were grown in medium containing 0.1% of BSA for additional 24h. Cells were then removed from plates with 0.01% trypsin and seeded on polycarbonate filters coated with 25μg/mL fibronectin. Cells were allowed to migrate for 6h (MCF-7 and MDA-MB-157) or 8h (BT-474 cells) in response to 10nM of insulin or IGF-2 added to the lower chamber. Values are mean±SEM of three independent experiments done in duplicate and are expressed as percent of untreated scramble cells (basal). **(e)** Colony formation. MCF-7, MDA-MB-157 and BT-474 breast cancer cells were transiently transfected as in **(a)**, were seeded in soft-agar, as described in Materials and Methods. Cells were plated in triplicate and cultured in serum free medium containing 2.5% CS-FCS for 3 weeks. Colonies developed only from MCF-7 and MDA-MB-157 cells and not from BT-474. Colonies were stained with MTT and then photographed. The histogram represents the mean number of colonies shown in **(e)**. Error bars indicate SEM (n = 3 wells). **(b-e)** NS, *0.01 < p < 0.05; p > 0.05; **0.001 < p < 0.01; ***p < 0.001; statistical significance was calculated using one-way ANOVA followed by Bonferroni test.

We next evaluated whether DDR1 expression could modulate the biological effects of insulin and IGF-2 in breast cancer cells. Indeed, in all three breast cancer cell lines, MCF-7, MDA-MD-157 and BT-474, DDR1 silencing by a pool of four specific siRNA oligos (Figure 3a) was associated with marked inhibition of cell viability as assessed by the MTT assay (Figure 3b) and cell cycle progression (Figure 3c), both in unstimulated cells and after treatment with either insulin or IGF-2. DDR1 silencing additionally inhibited other protumorigenic actions of insulin and IGF-2, such as cell invasion through fibronectin-coated filters (Figure 3d) and colony formation in semi-solid agar (Figure 3e). Accordingly, transient DDR1 overexpression (Figure 4a) enhanced cell viability (Figure 4b), cell cycle progression (Figure 4c), cell invasion (Figure 4d), and colony formation (Figure 4e), both in unstimulated cells and after stimulation with either insulin or IGF-2. In our experimental conditions, BT-474 cells did not form colonies in soft agar with charcoal-stripped serum-containing media.

Noteworthy, all these experiments were carried out in the absence of collagen, indicating that this modulation of insulin and IGF-2 effects is independent of DDR1 function as a collagen receptor.

### DDR1 is a critical regulator of IR expression and downstream signaling

**Figure 5:**
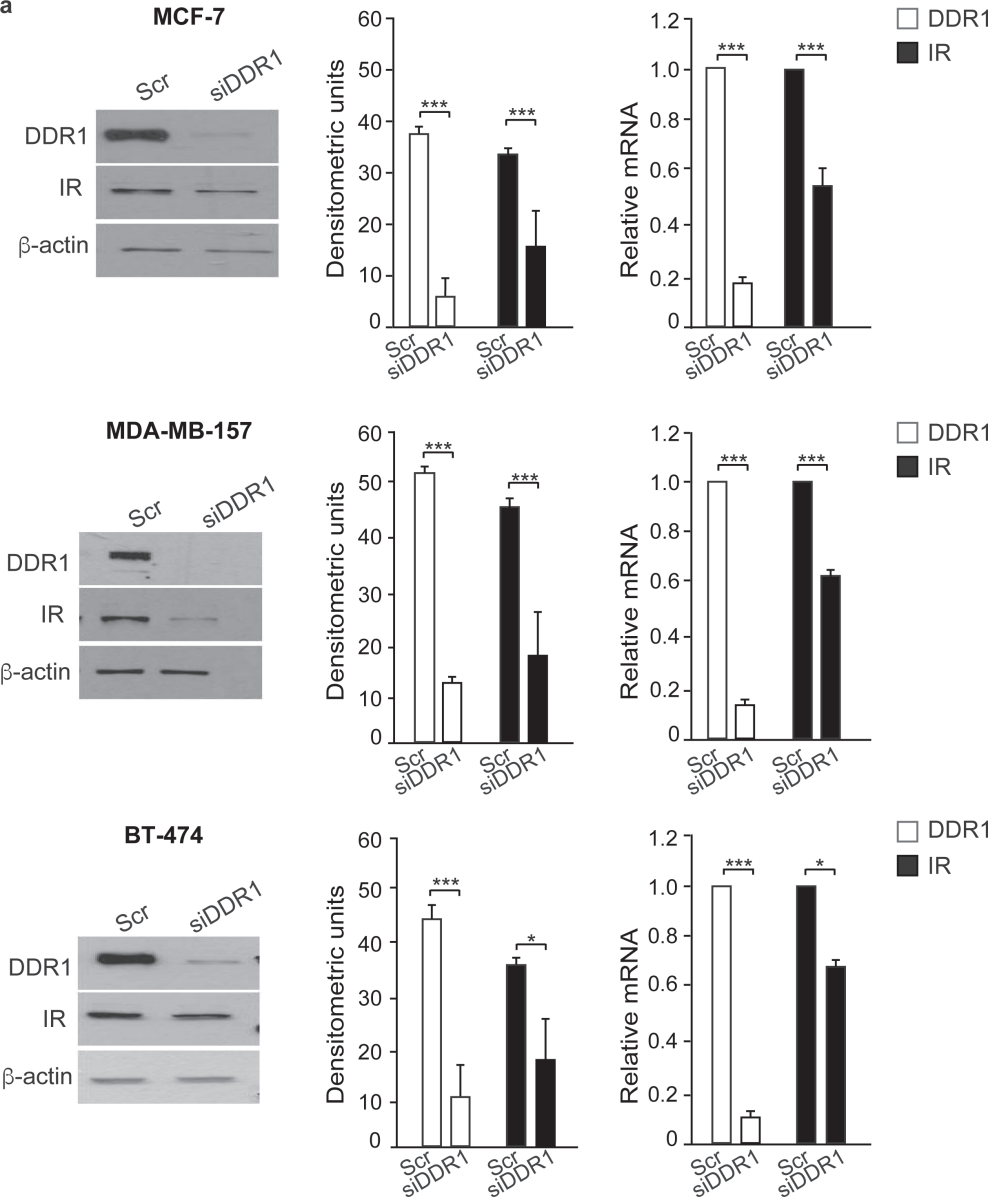

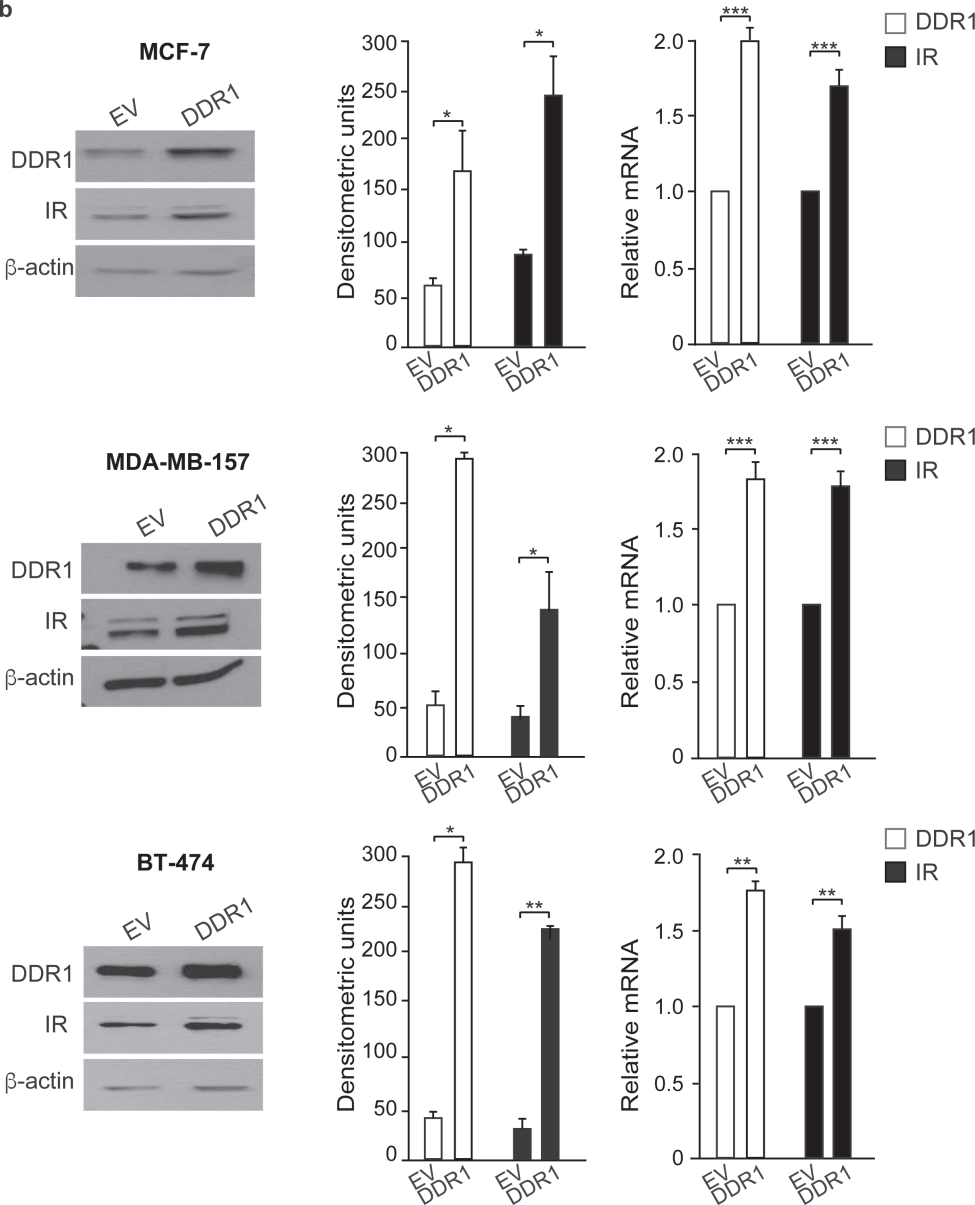
DDR1 affects IR expression **(a)** IR protein and mRNA expression in DDR1-depleted cells. MCF-7, MDA-MB-157 and BT-474 breast cancer cells were transiently transfected with either a pool of four scramble or four DDR1 siRNA oligos. After 48h, cells were lysed and analyzed by SDS-PAGE and immunoblotted with the indicated primary antibodies. β-actin was used as control for protein loading. Blot is representative of three independent experiments. The histograms represent the mean±SEM of densitometric analysis after normalization against β-actin. In the same transfected cell lines, IR mRNA levels were evaluated by qRT-PCR analysis and values were normalized using human β-actin as housekeeping control gene. In parallel, DDR1 mRNA was evaluated by qRT-PCR to confirm DDR1 depletion (graphs on the right). **(b)** IR protein and mRNA expression after DDR1 overexpression. Breast cancer cells were transiently transfected with either constitutive empty (pCMV6-EV) or human DDR1 (pCMV6-DDR1) expressing vectors. After 48h, cells were lysed, analyzed by SDS-PAGE and immunoblotted with the indicated primary antibodies. β-actin was used as control for protein loading. The top panels show a representative blot of three independent experiments. The histograms represent the mean±SEM of densitometric analysis after normalization over β-actin. In the same transfected cells, IR mRNA levels were evaluated by qRT-PCR analysis and values were normalized using human β-actin as housekeeping control gene. The overexpression of DDR1 was confirmed measuring DDR1 mRNA by qRT-PCR (graphs on the right). **(a-b)** *0.01 < p < 0.05; *0.01 < p < 0.05; **0.001 < p < 0.01; ***p < 0.001; (scramble *vs*. siDDR1 and EV *vs*. DDR1 and transfected cells). statistical significance was calculated using Student's t-test.

**Figure 6:**
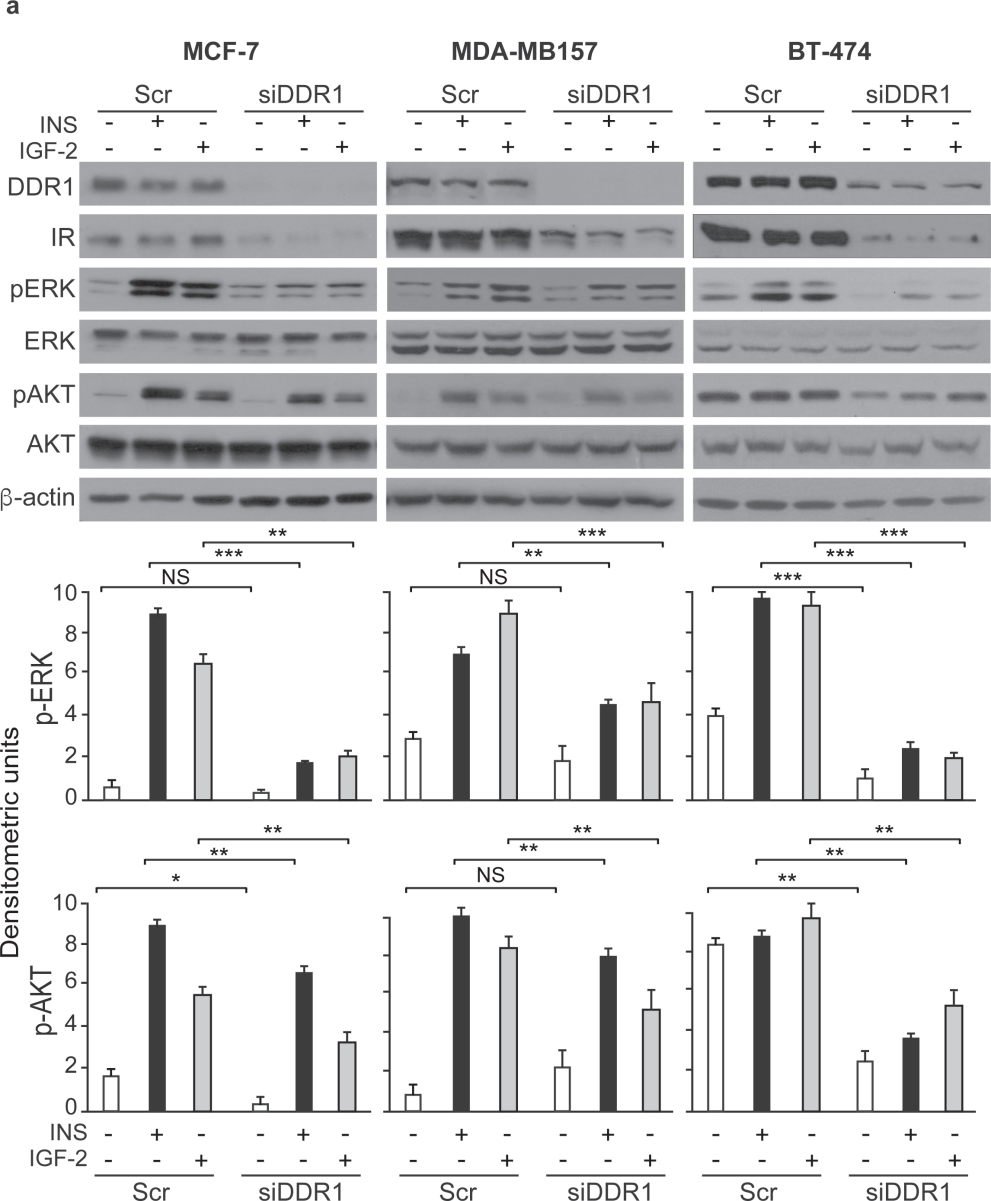

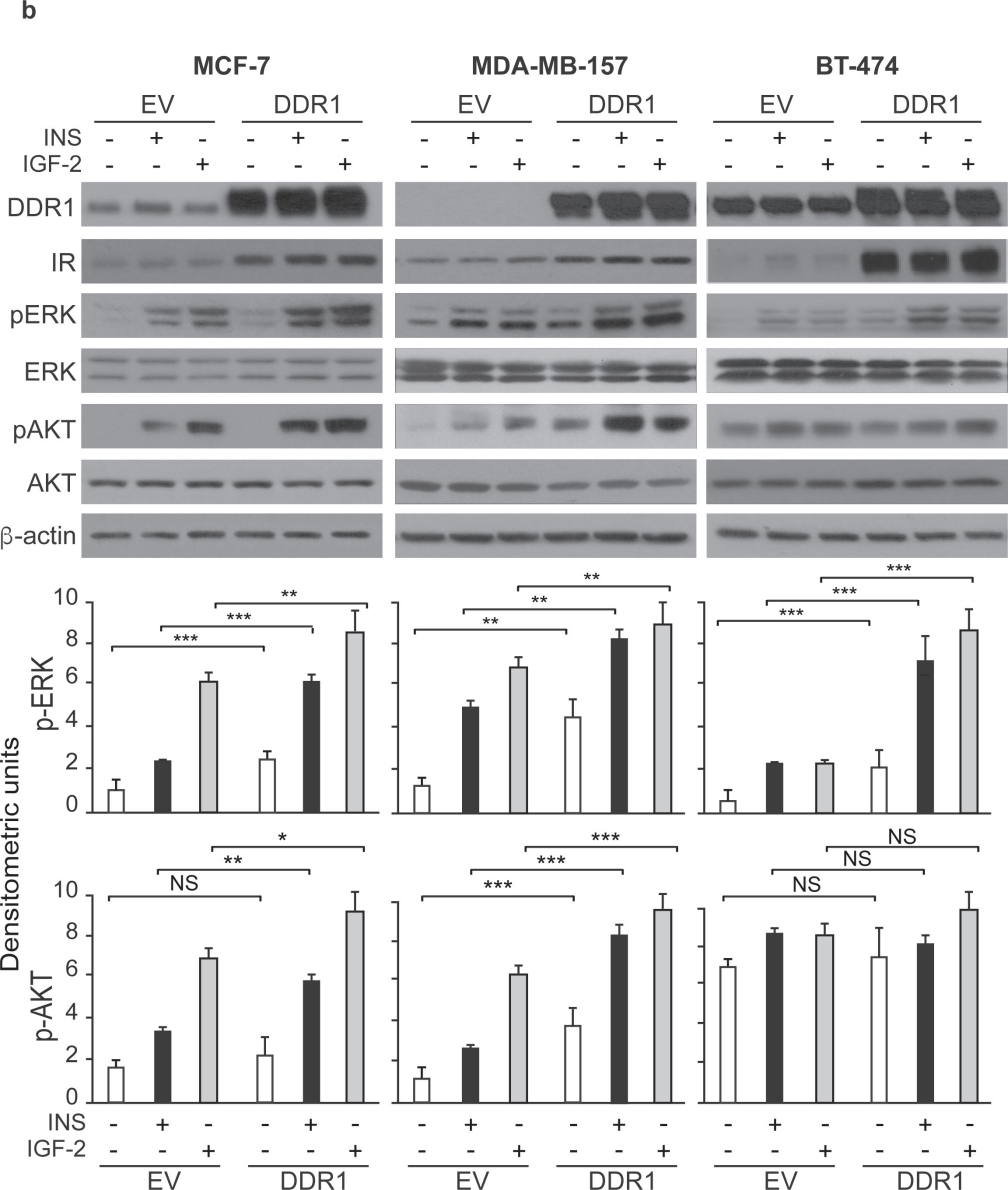
DDR1 depletion or overexpression affects insulin and IGF-2 downstream signaling in human breast cancer cells **(a)** Insulin and IGF-2 signaling after DDR1 depletion. MCF-7, BT-474 and MDA-MB-157 breast cancer cells were transiently transfected with a pool of four scramble or of four siRNA oligos against DDR1. After 48h, cells were grown in medium containing 2.5% of CS-FCS for 24h and then stimulated with or without 10nM of insulin or IGF-2 for 5 minutes. Cells were then lysed and analyzed by SDS-PAGE and immunoblotted with the indicated primary antibodies. β-actin was used as control for protein loading. The top panels show a representative of three experiments. The histograms represent the mean±SEM of densitometric analysis of three independent experiments after normalization of each phosphoprotein against β-actin. **(b)** Insulin and IGF-2 signaling after DDR1 overexpression. MCF-7, BT-474 and MDA-MB-157 breast cancer cells were transiently transfected with plasmids encoding either the human DDR1 cDNA (pCMV6-DDR1) or the corresponding empty vector (pCMV6-EV). After 48h, cells were grown in medium containing 2.5% of CS-FCS for 24h and then stimulated with or without 10nM of insulin or IGF-2 for 5 minutes. The activation of downstream signaling was assessed as in **(a)**. Blots are representative of three independent experiments. The histograms represent the mean±SEM of densitometric analysis of three independent experiments after normalization of each phosphoproteins against β-actin. **(a-b)** NS, p > 0.05; *0.01 < p < 0.05; **0.001 < p < 0.01; ***p < 0.001; statistical significance was calculated using one-way ANOVA followed by Bonferroni test.

As DDR1 regulates IR biological actions, we evaluated whether DDR1 regulated IR expression levels and/or IR intracellular signaling after ligand stimulation. Indeed, DDR1 silencing by a pool of four specific siRNA oligos significantly reduced the expression of IR protein and mRNA (Figure 5a) in all three cell lines tested. Conversely, transient DDR1 overexpression using a constitutive DDR1 encoding vector (pCMV6-DDR1) resulted in significant increase of IR protein and mRNA levels (Figure 5b). The increase in IR protein expression was further confirmed transiently transfecting MCF-7, MDA-MB-157 and BT-474 cell lines with pTZ doxy-inducible lentiviral vector encoding for DDR1 (Supplementary Figure 1). Accordingly, IR downstream signaling in response to insulin and IGF-2 stimulation was inhibited by DDR1 silencing (Figure 6a) and increased by DDR1 overexpression (Figure 6b). In particular, the two main IR downstream signaling cascades, the AKT and ERK1/2 pathways, were significantly affected by changes in DDR1 expression (Figure 6a and 6b).

Taken together these data indicate that DDR1 modulates IR response to insulin and IGF-2 by regulating IR expression levels.

### DDR1 regulates IR expression at multiple levels

**Figure 7:**
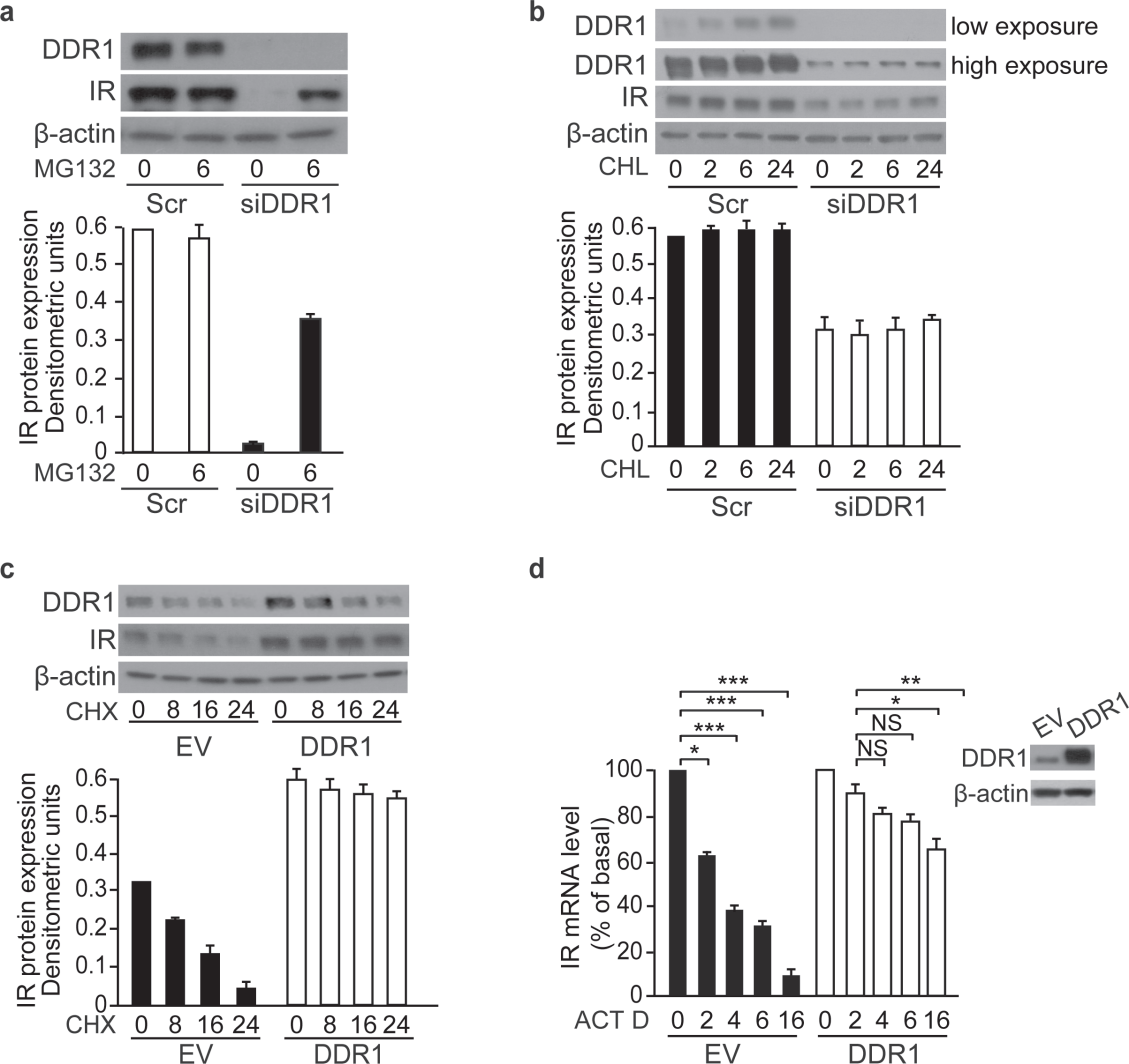
DDR1 stabilizes IR both at the protein and mRNA level in MCF-7 cells **(a)** DDR1 depletion affects IR proteasomal degradation. MCF-7 cells were transiently transfected with either a pool of four scramble or four DDR1-specific siRNA oligos. After 48h, cells were treated with 1μM of the proteasome inhibitor MG132 for 6h. **(b)** DDR1 depletion does not affect IR lysosomal degradation. MCF-7 cells were transiently transfected as in **(a)**. After 48 h, cells were treated with 10μM of the cloroquine (CHL) for 2, 6, and 24h. **(c)** Cycloheximide treatment in DDR1 overexpressing cells. MCF-7 cells were transiently transfected with either pCMV6-EV or pCMV6-DDR1 encoding vectors. After 24 h, cells were treated with 100 μM of cycloheximide (CHX) for 8, 16, and 24h. **(d)** Actinomycin D treatment in DDR1 overexpressing cells. MCF-7 cells were transiently transfected as in **(c)**. After 24h, cells were treated with 2.5μg/mL of actinomycin D (ACT D) for 2, 4, 6, and 16h. IR mRNA levels were evaluated by qRT-PCR analysis and values were normalized using human β-actin as housekeeping control gene. DDR1 overexpression was confirmed by western blot analysis as shown on the right of the histogram. **(a-c)** Samples were analyzed by western blotting for DDR1 and IR expression. β-actin antibody was used as control. A representative blot of three independent experiments is shown. The histogram represents the mean±SEM of densitometric analysis after normalization against β-actin. **(a-d)** NS, p > 0.05; *0.01 < p < 0.05; **0.001 < p < 0.01; ***p < 0.001. Statistical significance was calculated using Student's t-test.

As DDR1 enhanced both IR mRNA and protein expression levels, we explored the mechanisms responsible for this effect. We first evaluated 26S proteasome-dependent IR protein degradation. We found that the decrease in IR protein levels induced by DDR1 silencing was partially reversed after treating MCF-7 cells with the 26S proteasome inhibitor, MG132 (Figure 7a). Next, we tested the contribution of the lysosomal degradation pathway by using the lysosomal inhibitor cloroquine. We found that, DDR1 levels were affected by cloroquine treatment in scramble-transfected cells, but not after DDR1 silencing (Figure 7b). Taken together, these results indicate that DDR1 affects the intracellular pool of IR protein degraded *via* proteasome but not by lysosomal degradation.

As DDR1 additionally affected the steady-state level of IR mRNA (Figure 5a and 5b), we asked whether translational and/or transcriptional mechanisms were involved. To this aim we studied MCF-7 cells at different time points after treatment with either the protein synthesis inhibitor cycloheximide or the transcriptional inhibitor actinomycin D. We found that cycloheximide significantly prolonged IR protein half-life in cells transiently overexpressing DDR1 as compared to control cells (EV transfected). Indeed, in DDR1 overexpressing cells IR up-regulation was not affected by 24h of cycloheximide treatment; in contrast, in control cells IR protein levels decreased to roughly 50% after 16h exposure to cycloheximide, suggesting that DDR1 increases IR protein by additionally affecting its *de novo* synthesis (Figure 7c). Similarly, actinomycin D treatment reduced IR mRNA more markedly in control cells (EV) than in DDR1-transfected cells; after 4h exposure to the drug IR mRNA levels were 60% of basal in EV transfected cells vs. 20% in DDR1 overexpressing cells (Figure 7d).

**Figure 8:**
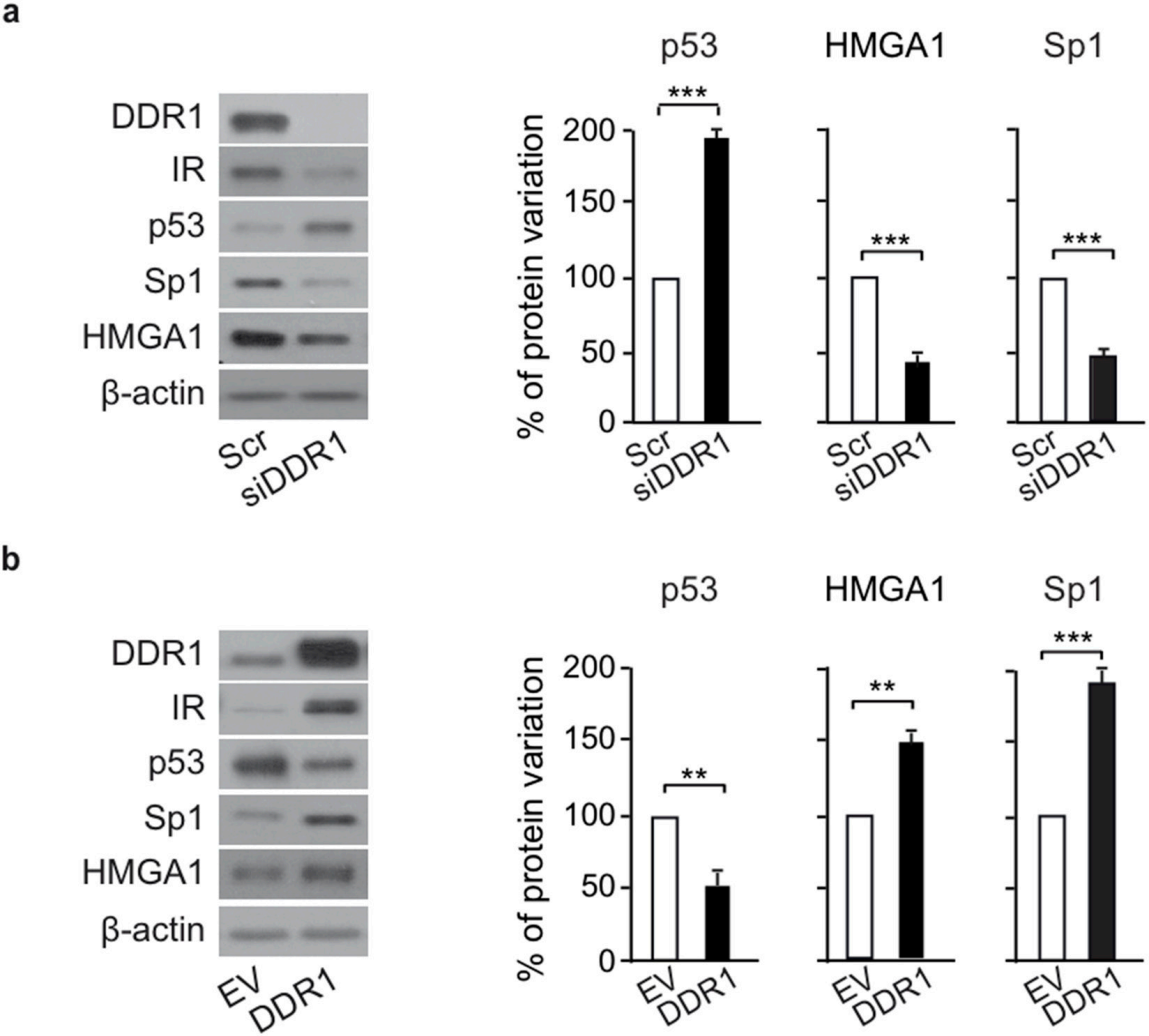

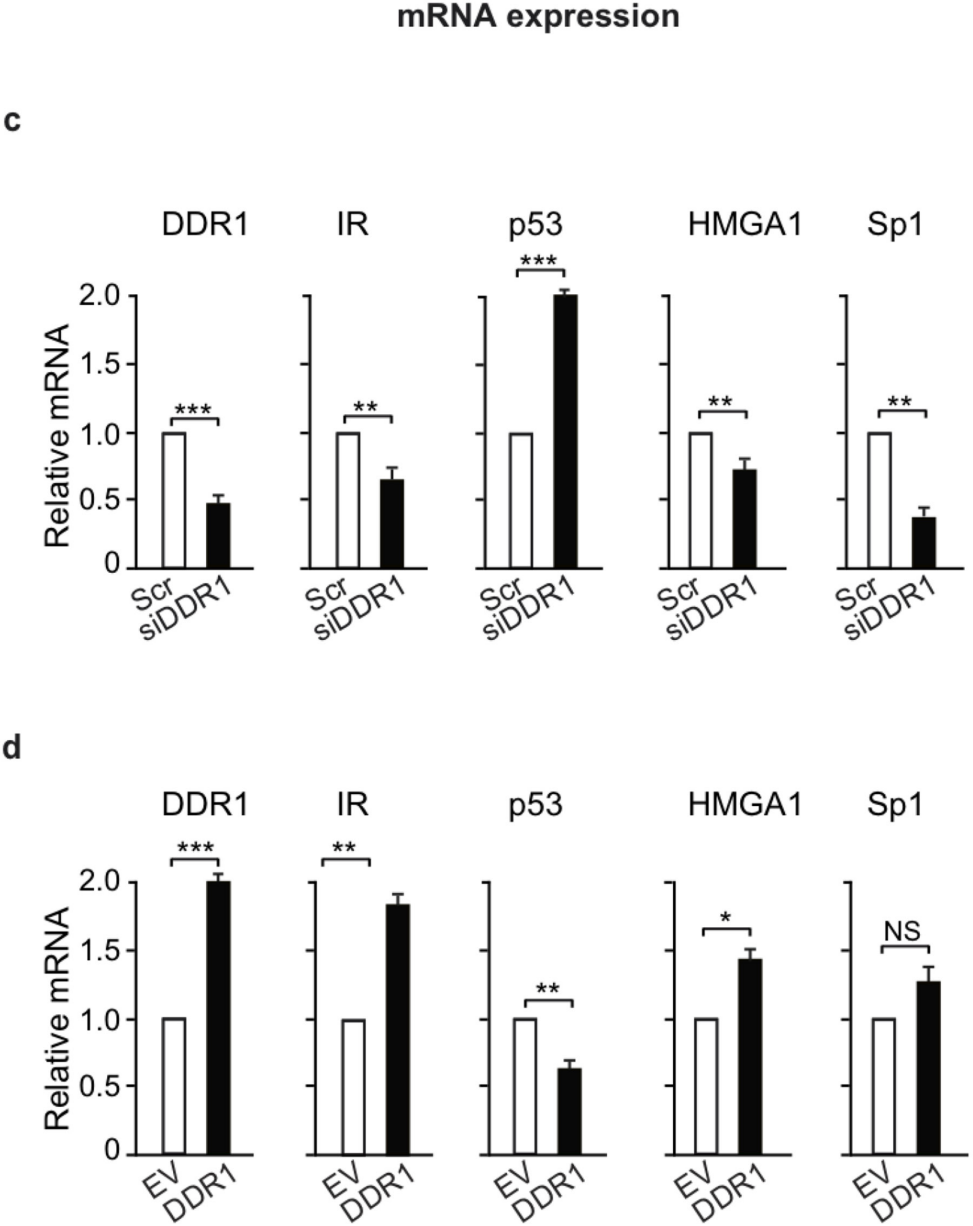
DDR1 affects IR – dependent transcription factors **(a)** Protein levels of IR-dependent transcription factors after DDR1 depletion. MCF-7 breast cancer cells were transiently transfected with either a pool of four scramble or four DDR1 specific siRNA oligos. After 48h, protein expression of IR, and of p53, HMGA, and Sp1 transcription factors was evaluated by western blotting. The histograms represent the mean±SEM of densitometric analysis of three independent experiments after normalization against β-actin. **(b)** Protein levels of IR-related transcription factors after DDR1 overexpression. MCF-7 cells were transiently transfected with either either pCMV6-EV or pCMV6-DDR1 encoding vectors. After 48h, protein expression of IR, p53, HMGA, and Sp1 was evaluated by western blotting. The histograms represent the mean±SEM of densitometric analysis of three independent experiments after normalization against β-actin. **(c)** mRNA expression of IR-related transcription factors after DDR1 depletion. MCF-7 cells transfected as in **(a)** were evaluated by qRT-PCR analysis for IR, p53, HMGA, and Sp1 mRNA levels. Values were normalized using human β-actin as housekeeping control gene. DDR1 mRNA levels were also evaluated as control for DDR1 depletion efficiency. Values represent the mean±SEM of three independent experiments. **(d)** mRNA expression of IR-related transcription factors after DDR1 overexpression. In cells transfected as in **(b)**, IR, p53, HMGA, and Sp1 mRNA levels were evaluated by qRT-PCR analysis and values were normalized using human β-actin as housekeeping control gene. DDR1 mRNA levels were also evaluated as silencing control. Values represent the mean±SEM of three independent experiments. **(a-d)** NS, p > 0.05; *0.01 < p < 0.05; **0.001 < p < 0.01; ***p < 0.001. Statistical significance was calculated using Student's t-test.

We next evaluated whether DDR1 could affect the main factors involved in the regulation of *IR* gene transcription, namely Sp1 and HMGA1, that are positive regulators, or p53, that is instead a negative regulator [26, 27]. Using MCF-7 cells, we found that IR down-regulation observed after DDR1 silencing was associated with significant reduction of Sp1 and HMGA1 proteins, and increased levels of p53 protein (Figure 8a). Conversely, IR upregulation observed after DDR1 transient overexpression was associated with opposite changes in Sp1, HMGA1 and p53 proteins (Figure 8b). The mRNA levels of these factors changed according to their protein expression profile both after DDR1 silencing (Figure 8c) or overexpression (Figure 8d). These data implicate DDR1 as a regulator of key transcription factors involved in IR gene expression.

Taken together, all these data indicate that DDR1 regulates IR expression at multiple levels by modulating protein degradation and stability, gene transcription and post-transcriptional mRNA regulation.

### DDR1 and IR are positively correlated in human breast cancer specimens

In order to verify whether DDR1 and IR expression positively correlated in human breast cancer specimens we used a bioinformatic analysis. By computing Pearson correlation between three probes for DDR1 and IR, we obtained 228 Pearson values out of 288 possible combinations (60 Pearson values were not calculated because the probes were lacking for some datasets) (Supplementary Table 1). We found 99 statistically significant (p-value ≤0.05) positive Pearson values and 6 negative Pearson values. Interestingly, 6 datasets (gse21618, gse42568, gse12276, gse21653, gse29271, gse76124) showed a persistent positive correlation between DDR1 and IR with all combinations of probes analyzed. Definitively, these computational data strongly suggested that a general positive correlation between DDR1 and IR transcripts exists in breast carcinoma (Supplementary Table 1).

**Figure 9:**
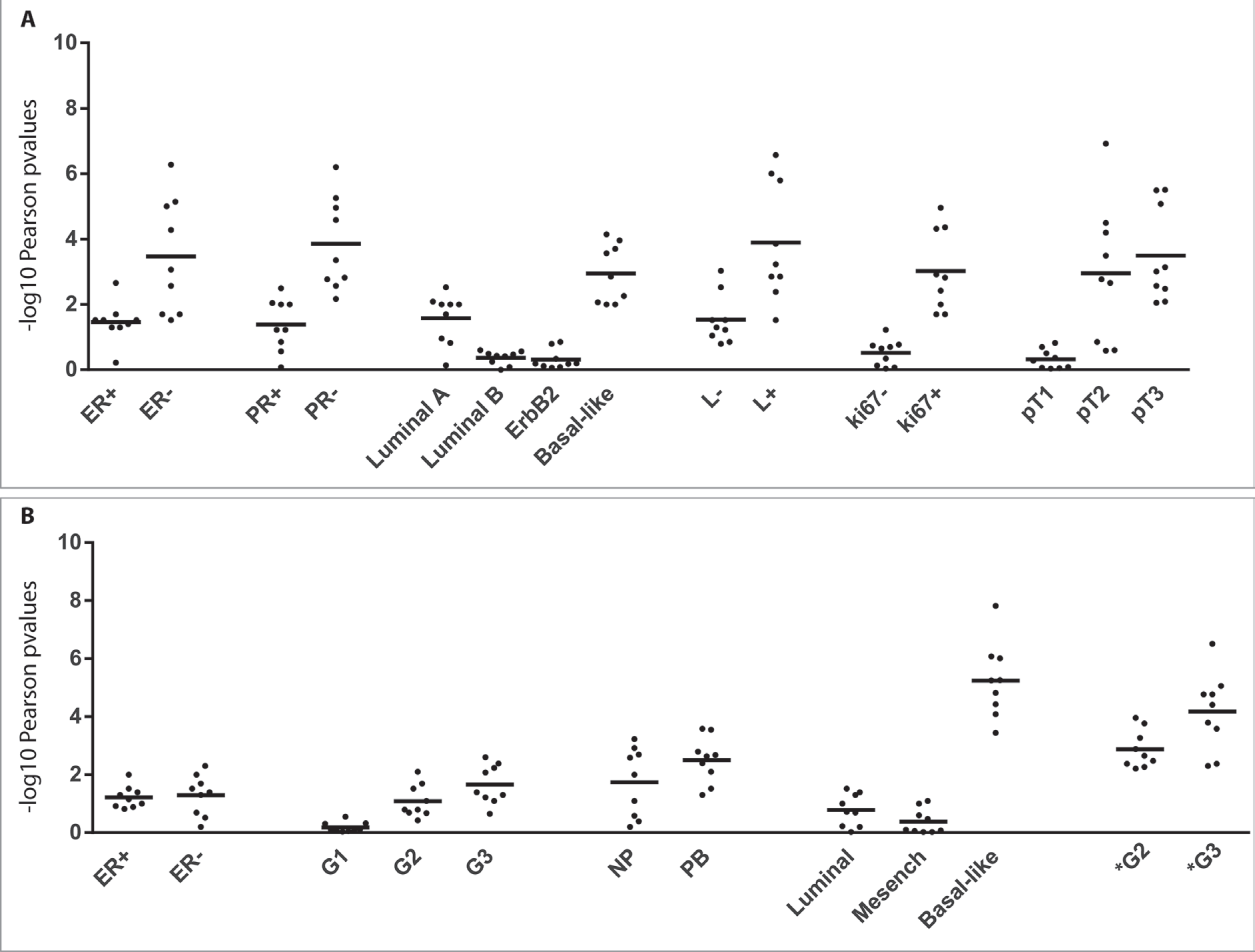
DDR1 correlates with IR in human breast cancer datasets Stratification analysis of DDR1 and IR correlative expression was performed. Pearson tests were calculated by separating the patients in clinical classes. This analysis was computed for GEO datasets gse21653 (Panel **A**), gse42568, gse76124 (Panel **B**). Black dots showed nine different combinations of Pearson test for three DDR1 probes (1007_s_at, 210749_x_at, 207169_x_at) and three IR probes (213792_s_at, 226450_at, 226216_at). Values plotted in the histograms are shown as –log10 of Pearson p-values. ER: estrogen receptor status; PR: progesterone receptor status; L: pathological lymph node status; Ki67: Ki-67 protein status evaluated by immunohistochemistry; pT: pathological tumor size; NB: normal breast tissue; PB: pathological breast tissue; G: grading of gse42568 datasets. G* grading of gse76124 datasets.

For GEO datasets gse42568, gse21653, gse76124 we found clinical and biological information useful to stratify the data, in order to verify whether DDR1-IR correlation is associated with bio-pathological features of patients (Figure 9A and 9B). This analysis showed that correlation of expression was more statistically significant in tumors with the following characteristics: 1) negative for ER; 2) negative for progesterone receptor; 3) basal-like histology; 4) positive for pathological lymph nodes; 5) positive for Ki-67 protein status; 6) pathological tumor size 3 (pT3); 7) grade 3 (G3). The correlations were statistically significant in both normal and tumor samples but p-values were considerably lower in the latter, especially in poorly differentiated tumors (Figure 9B).

## DISCUSSION

Our present results demonstrate that in human breast cancer cells DDR1 is a signaling partner of the IR and a strong modulator of IR expression and biological responses. DDR1 and IR co-localize and co-immunoprecipitate, especially after stimulation with either insulin or IGF-2. We also show that, after stimulation with insulin or IGF-2, DDR1 is rapidly internalized with the IR and co-localizes with the IR in the cytoplasm. These findings are reminiscent of similar data we previously observed with the IR-homolog IGF-1R [23]. Noteworthy, it has been demonstrated that DDR1 is internalized after binding to different forms of collagen (collagen I, IV and VI) [16, 18, 28]. Herein, we found that DDR1 co-internalization with IR is not dependent on collagen.

Remarkably, DDR1 modulates the two main signaling cascades downstream of the IR. DDR1 silencing significantly reduced the phosphorylation of both AKT and ERK1/2 in response to insulin and IGF-2, while DDR1 overexpression enhanced it. According to these findings, DDR1 enhanced breast cancer cell proliferation, invasion and colony formation in response to insulin and IGF-2. Moreover, IR silencing abrogated the effect of DDR1 overexpression on cell viability and invasion, not only in response to insulin and IGF-2 but also in unstimulated cells, indicating that IR expression was pivotal for the effects of DDR1.

We found that a major mechanism by which DDR1 affects intracellular signaling and biological responses to insulin and IGF-2 is the modulation of IR expression. DDR1 affected IR expression at multiple levels by regulating both IR protein and mRNA half-life as well as transcription factors known to modulate IR gene expression [26, 27].

The decrease in IR protein levels induced by DDR1 depletion was sensitive to inhibitors of the proteasome but not to lysosomal inhibition. Collectively, these results suggest that DDR1 may preferentially regulate the intracellular pool of IR protein degraded via the proteasome, but it does not affect IR turnover dependent on lysosomal degradation.

Ubiquitination of tyrosine-kinase receptors, including the IGF-1R and IR [29–34], plays an important role in regulating ligand-dependent receptor endocytosis and sorting for degradation. However, whether DDR1 expression may affect IR ubiquitination remains to be elucidated. Further studies are required to fully clarify the molecular mechanisms associated with DDR1 action on IR trafficking and gene expression regulation.

IR overexpression and in particular IR-A isoform upregulation, as well as hyperinsulinemia and IGF-2, have a well-established role in breast cancer both *in vitro* and *in vivo* [1, 35–39]. Our data strongly suggest that DDR1 might be an important determinant of IR overexpression in breast cancer. Significantly, we have previously shown that IGF-1, IGF-2 and insulin induce DDR1 upregulation in breast cancer cells by activating the AKT/miR199a-5b pathway [24]. Others have shown that DDR1 is one of the prominent molecules expressed by sarcomas characterized by constitutive IGF-2 overexpression [40]. Thus, the IR–DDR1 crosstalk constitutes a positive feedback loop enhancing the effects of insulin and IGF-2 in breast cancer cells.

These findings are consistent with previous data indicating that DDR1 as well as the IGF-2/IR-A loop are important regulators of prenatal growth [19] and cancer progression [20, 41].

By analyzing publicly available databases, we found that the correlation between DDR1 and IR is stronger in breast cancer specimens than in normal breast tissues. Importantly, the correlation between DDR1 and IR expression levels is even stronger in breast cancers with aggressive characteristics, such as basal-like phenotype, absence of estrogens and progesterone receptors, metastatic lymph-nodes, elevated Ki67 staining and tumor grading, confirming that this functional crosstalk might have important clinical implications in breast cancer. In this regard, our present finding that DDR1 affects both the AKT and the ERK1/2 pathways is particularly relevant, as IR/IGF-1R inhibitors proposed for cancer treatment are generally more effective in blocking the AKT pathway than inhibiting the ERK pathway, and may actually stimulate this pathway in certain models [42–44]. Thus, DDR1 may work as a possible novel candidate in targeting the IGF-2/IR-A loop in cancer.

In summary, this study demonstrates that DDR1 associates with the IR, enhances the activation of IR downstream signaling and the mitogenic and pro-invasive effects of insulin and IGF-2 in breast cancer cells. DDR1 emerges as a novel and important regulator of IR expression by acting at multiple levels. All these effects are distinct from the previously characterized role of DDR1 as a collagen receptor. In accordance with these *in vitro* findings, DDR1 and IR expression levels are strongly correlated in breast cancer specimens, and especially in cancers with aggressive characteristics, suggesting that the DDR1 - IR axis could be a valuable therapeutic target in human breast cancer.

## MATERIALS AND METHODS

### Materials

Insulin and IGF-2 were purchased from Prepotech (Rocky Hill, NJ); bovine serum albumin (BSA), fibronectin, collagen Type IV, actinomycin D, cycloheximide, cloroquine, and MG132 from Sigma-Aldrich (Saint Louis Missouri, USA); Metafectene PRO from Biontex Laboratories GmbH (Germany); lipofectamine RNAiMax, Opti-MEM, fetal calf serum (FCS), Geneticin (G-418), puromycin, TRIzol Reagent, ThermoScript RT kit, SYBR Green MasterMix from Life Technologies, Inc. Laboratories (Paisley, UK); MTT, nitrocellulose membranes, HRP-conjugated secondary antibodies from Amersham Biosciences (Little Chalfont, UK).

Constructs encoding either an empty vector (pCMV6- EV) or the human wild type DDR1 isoform a (DDR1wt) cDNAs were from OriGene (Rockville, MD, USA). The specific silencer Select Pre-designed pool of four siRNA oligos for DDR1 (Human DDR1 siGENOME SMARTpool Cat M-003111–04) and the negative control, consisting of a pool of four scramble siRNAs were from Thermo Fisher Scientific Dharmacon (NYSE:TMO). Doxycicline inducible lentivirus vectors (pTZ) encoding for DDR1 cDNA or control vector GFP were purchased from GE Healthcare Dharmacon Europe.

### Cell cultures

The human cancer cell lines MCF-7, BT-474, MDA-MB-157, T47D, ZR-75, MDA-MB-231, MDA-MB-468 were purchased from the American Cell Type Culture Collection and cultured according to the manufacturer's instructions. Cells were grown in Medium supplemented with 10% fetal bovine serum (FBS).

### Western blot analysis

Cell lysates were subjected to Western blot analysis as previously described [45]. The following antibodies were used: anti-DDR1 (C-20), anti-IRβ (C-19), anti-p53 (DO-1), anti HMG-I/HMG-Y (FL-95) (Santa Cruz Biotechnology); anti-p-Akt (Ser473), anti-AKT, anti-p-ERK1/2 (T202/Y204), anti-ERK1/2, anti-Sp1 (D4C3) (Cell Signaling Technology); anti-β-actin (Sigma-Aldrich, Saint Louis Missouri, USA).

### Real-time PCR

Total cellular RNA was extracted using TRIzol Reagent according to the manufacturer's protocol, as previously described [46]. qRT–PCR was used to confirm the expression levels of mRNAs. Total RNA (2μg) was reversely transcribed using the ThermoScript RT kit and oligo(dT) primers. Synthesized cDNA was combined in a qRT-PCR reaction using primers for the gene of interest (Table 1). The ΔΔCt method of relative quantification and SYBR Green chemistry were used to measure mRNA.

**Table 1:** Primers used for quantitative PCR

h-DDR1 total	FW 5′-GCGTCTGTCTGCGGGTAGAG-3′ RV 5′-ACGTCAGATAAATACATTGTCT-3′
h-IR	FW 5′-CGTGGAGGATAATTACATCGTGTT-3′ RV 5′-TGGTCGGGCAAACTTTCTG-3′
p53	FW 5′-CACTGCCCAACAACACCAGCTCCT-3′ RV 5′-GTCTGAGTCAGGCCCTTCTGTCTT-3′
HMGA1a	FW 5′-AGGAAAAGGACGGCACTGAGAA-3′ RV 5′-CCCCGAGGTCTCTTAGGTGTTGG-3′
Sp1	FW 5′-TGAAAAAGGAGTTGGTGGC-3′ RV 5′-TGCTGGTTCTGTAAGTTGGG-3′
h β-actin	FW 5′-GACAGGATGCAGAAGGAGATCACT-3′ RV 5′-TGATCCACATCTGCTGGAACC T-3′

Fw: forward; Rv: reverse

### Confocal microscopy

MCF-7 cells were plated onto coverslips, serum starved for 24h, and then stimulated with either insulin or IGF-2. Coverslips were processed for immunofluorescence and confocal analysis at the Sidney Kimmel Cancer Center Bioimaging Core Facility, as previously described [23]. Primary antibodies used were: anti-IR (polyclonal antibody C-19, Santa Cruz Biotechnology), anti-DDR1 (polyclonal antibody C-20, Santa Cruz Biotechnology). After incubation with primary antibodies, the coverslips were incubated using secondary antibodies, goat anti-mouse IgG Alexa Fluor 488 (Invitrogen) and goat anti-rabbit IgG Alexa Fluor 594 (Invitrogen). Coverslips were analyzed and photographed on a Nikon AIR inverted confocal microscope with a Plan-Apo 60x oil immersion lens at room temperature and NIS Elements C software. Images were analyzed using Image J. Pictures are representative of at least 10 independent fields from three independent experiments. Fields were selected for the presence of cells with the following criteria: well defined limits, clear identification of nucleus and absence of intersection with neighboring cells. An average of 100 cells was examined for each condition. Data are representative of ~80% of the total number of cells examined. Co-localization index was calculated using NIH ImageJ software.

### Immunoprecipitation analysis

Cells were lysed and processed as previously described [49]. The following antibodies were used for Immunoprecipation: anti-DDR1 (monoclonal antibody MAB2396, R&D System) and anti-HA.11 (monoclonal antibody 16B12, Covance). The following antibodies were used for Western Blotting: anti-DDR1 (policlonal antibody C-20, sc-532) and anti-IR (polyclonal antibody C-19, Santa Cruz biotechnology).

### Gene silencing by small interfering RNA, and gene overexpression

For small interfering RNA (siRNA) experiments, cells were transiently transfected with a mixture containing OptiMem, Lipofectamine RNAiMax and either a pool of four scramble siRNA oligos (10nM) or a pool of four specific siRNA oligos for DDR1 (10nM).

For overexpression experiments, cells were transiently transfected with a mixture containing Opti-Mem, Metafectene PRO and the DNA of interest or the corresponding control empty vectors.

Most experiments of DDR1 overexpression were performed by transiently transfecting cells with plasmids encoding either the human constitutive pCMV6-DDR1 or the corresponding empty vector (pCMV6-EV). Cells were then processed after 48h, according to the aim of the experiment. As, a complementary approach to overexpress DDR1, cells were transiently transfected with a doxy-inducible pTZ-DDR1 lentiviral vector or control pTZ-GFP vector. Twenty-four hours after transfection, cells were incubated with doxycycline (1μg/mL) for 48h and then processed to perform the experiments of interest.

To evaluate the IR downstream signaling, transfected cells were serum starved for 24h, and stimulated with insulin (10nM) or IGF-2 (10nM) for 5 min.

### Cell viability

Cell viability was measured by the methyl thiazolyl tatrazolium (MTT) test. MCF-7, MDA-MB-157 and BT-474 cell lines were plated in 48-multiwell plates under standard culture conditions. After 24h, cells were transfected with a pool of four DDR1 siRNA oligos or DDR1 expressing vectors and the relative negative controls. After 24h, cells were serum-starved for 24h and then stimulated with insulin (10nM) or IGF-2 (10nM) for additional 48h. The cells were then incubated with medium containing 5mg/mL MTT and processed as previously described [47].

### Invasion assay

The ability of cells to invade the extracellular matrix was measured with Boyden's chamber technique as described [23]. Cells, serum starved for 24h, were placed on polycarbonate filters (8μm pore size, Corning Costar) coated on the upper side with 25μg/mL fibronectin. Filters were placed over bottom chambers containing serum-free medium with or without ligand (10nM). After incubation for 6-8h, depending on the cell type, cells on the upper surface of filters were removed with a cotton swab, and the filters were stained for 30min with crystal violet (0.05% crystal violet in PBS plus 20% ethanol). After three washes with water, crystal violet was solubilized in 10% acetic acid for 30min at room temperature, and its concentration was evaluated by absorbance at 595 nm.

### Cell cycle evaluation

Cells synchronized for 24h in serum-free medium were exposed to insulin (10nM) or IGF-2 (10nM) for 48h and subjected to fluorescence-activated cell sorting (FACS) analysis, as previously described [48].

### Soft-agar colony formation assay

Anchorage-independent growth was assessed as previously described [23] with some modifications. Briefly, a mixture of 0.66% agar and medium containing 2.5% of CS-FCS was plated on the bottom of each well plate (hard-agar). Then, cells suspended in 2.5% CS-FCS medium containing 0.33% agar (soft-agar) were plated on the top of the hard-agar layer. Top agar was then covered with culture medium with or without insulin (10nM) or IGF-2 (10nM). Stimulus was changed twice a week and cells were cultured for 3 weeks. Colonies were visualized with 0.5mg/mL MTT, photographed and analyzed with NIH ImageJ.

### Analysis of expression correlation

The correlation of expression between DDR1 and IR across 32 different human datasets of microarray experiments was analyzed on breast cancer biopsies and cell lines. We selected from Gene Expression Omnibus (GEO) (https://www.ncbi.nlm.nih.gov/geo) the Affymetrix GeneChip Human Genome U133A and U133 plus 2.0 arrays normalized by MAS5.0 algorithm. We computed the Pearson correlation and p-value between DDR1 and IR for each dataset considered. The Pearson value provides an index of positive expression correlation between two genes when it is positive, otherwise, negative expression correlation when it is negative. Multiple probes of DDR1 and IR transcripts were assayed in the Affymetrix chips analysis. Therefore, we separately calculated the Pearson value of three probes for DDR1 and three probes for IR. For GEO datasets showing a persistent statistically significant positive correlation between DDR1 and IR for each combination of probes, we performed a stratification analysis by separating the datasets into clinical categories and iterating the Pearson analysis. GEO ID of datasets and probes are reported in Supplementary Table 1.

### Densitometric and statistical analysis

Densitometry results were obtained by using NIH ImageJ. Differences between means were evaluated by one-way ANOVA followed by post-hoc analysis of significance (Bonferroni test) for the comparison between more than two groups, whereas the Student's *t* test for unpaired samples was used for comparisons between two groups. The level of significance was set at p<0.05. Statistical analysis was performed with GraphPad Prism6 (GraphPad Software, San Diego, CA). Data were expressed as mean±SEM.

## SUPPLEMENTARY MATERIALS FIGURES AND TABLES





## Abbreviations

IR: insulin receptor
IR-A: insulin receptor isoform A
DDR1: discoidin domain receptor 1
IIGFs: insulin/IGF signaling
IGF-1R: insulin growth factor receptor
IGF-1: insulin growth factor 1
IGF-2: insulin growth factor 2
EMT: epithelial mesenchymal transition
PTB: phosphotyrosine binding
ERK: extracellular signal-related kinase
3′UTR: 3′ untranslated region
HMGA1: high-mobility group protein
MTT: methyl thiazolyl tatrazolium
HRP: horseradish peroxidase
GFP: green fluorescent protein
FACS: fluorescence-activated cell sorting
FCS: fetal calf serum
