# Supplementary material for: Discoidin domain receptor 1 modulates insulin receptor signaling and biological responses in breast cancer cells
Source: Oncotarget. 2017 May 19;8(26):43248–70. doi: 10.18632/oncotarget.18020 (PMC5522143; doi:10.18632/oncotarget.18020)
Supplement: Supplementary file 1 [file oncotarget-08-43248-s001.pdf]

Discoidin domain receptor 1 modulates insulin receptor signaling and biological responses in breast cancer cells

SUPPLEMENTARY MATERIALS

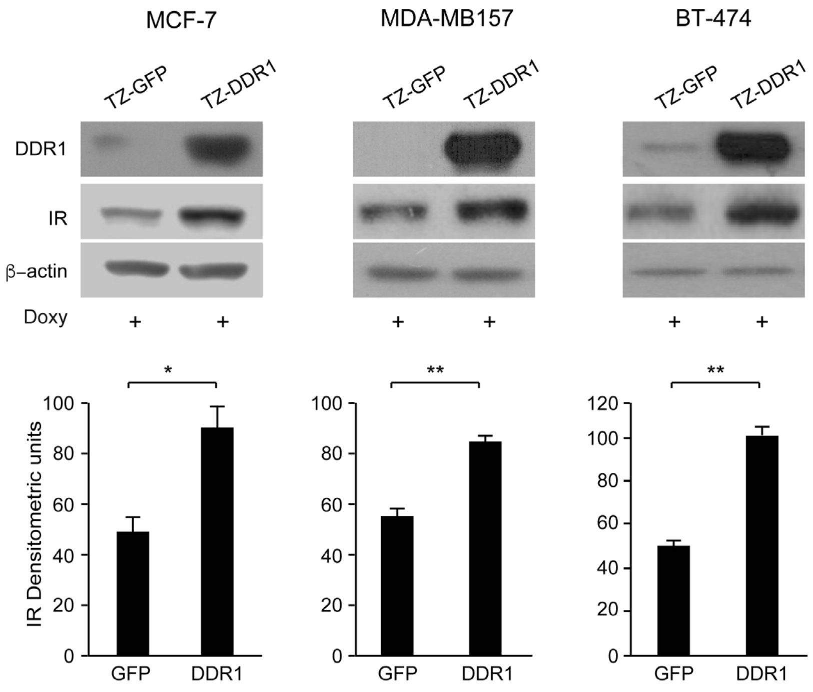

Supplementary Figure 1: DDR1 overexpression increases IR expression in breast cancer cell lines. MCF-7, MDA-MB-157 and BT-474 cells were transiently transfected with pTZ doxy-inducible lentiviral vectors encoding for DDR1 or control GFP. Western blot analysis was performed upon 48h doxycycline treatment (1µg/mL). After 72h from transfection, cells were lysed, analyzed by SDS-PAGE and immunoblotted with the indicated primary antibodies. DDR1 induction was verified by immunoblotting using a specific primary antibody. β-actin was used as control for protein loading. Panels show a representative blot of two independent experiments. The histograms represent the mean±SEM of densitometric analysis after normalization over β-actin. NS, $p > 0.05$; *$0.01 < p < 0.05$; **$0.001 < p < 0.01$. Statistical significance was calculated using Student's t-test.

Supplementary Table 1: Correlation of expression between DDR1 and INSR

See Supplementary File 1
