# Supplementary material for: Discoidin domain receptor 1 modulates insulin receptor signaling and biological responses in breast cancer cells
Source: Oncotarget. 2017 May 19;8(26):43248–70. doi: 10.18632/oncotarget.18020 (PMC5522143; doi:10.18632/oncotarget.18020)
Supplement: Supplementary file 2 [file oncotarget-08-43248-s002.docx]

**Supplementary Table 1: Correlation of expression between DDR1 and INSR**

| **GEO ID** | **Sample number** | **C1** | **C2** | **C3** | **C4** | **C5** | **C6** | **C7** | **C8** | **C9** |
| --- | --- | --- | --- | --- | --- | --- | --- | --- | --- | --- |
| gse10797 | 66 | 0.128 | ND | ND | 0.088 | ND | ND | **0.293 (0.02)** | ND | ND |
| gse12093 | 136 | **0.44 (8.2e-08)** | ND | ND | **0.227 (7.8e-03)** | ND | ND | **0.174 (0.04)** | ND | ND |
| gse12276 | 204 | **0.235 (7.1e-04)** | **0.315 (4.5e-06)** | **0.168 (0.02)** | **0.242 (4.8e-04)** | **0.323 (2.4e-06)** | **0.174 (0.01** | **0.27 (9.3e-05)** | **0.334 (1.0e-06)** | **0.184 (8.5e-03)** |
| gse12777 | 51 | 0.145 | 0.195 | 0.069 | 0.175 | 0.236 | 0.089 | 0.116 | 0.161 | 0.052 |
| gse1456 | 159 | 0.132 | ND | ND | 0.001 | ND | ND | 0.101 | ND | ND |
| gse16391 | 55 | 0.01 | 0.073 | -0.015 | 0.045 | 0.059 | -0.06 | 0.047 | 0.056 | -0.035 |
| gse16446 | 120 | 0.151 | **0.196 (0.03)** | **0.318 (4.0e-04)** | 0.167 | 0.221 (0.02 | **0.387 (1.2e-05)** | **0.18 (0.05)** | **0.212 (0.02)** | **0.374 (2.6e-05)** |
| gse17785 | 12 | 0.437 | 0.494 | -0.074 | 0.436 | **0.597 (0.04 )** | -0.109 | 0.49 | 0.561 | -0.093 |
| gse2034 | 286 | **0.157 (7.9e-03)** | ND | ND | 0.115 | ND | ND | **0.158 (7.3e-03)** | ND | ND |
| gse2109 | 351 | **0.121 (0.02)** | **0.107 (0.05)** | **0.108 (0.049)** | 0.081 | 0.065 | **0.152 (4.2e-03)** | 0.086 | 0.104 | **0.16 (2.7e-03)** |
| gse21618 | 143 | **0.403 (6.0e-07)** | **0.443 (3.0e-08)** | **0.32 (9.9e-05)** | **0.277 (8.1e-04 )** | **0.356 (1.3e-05)** | **0.239 (4.1e-03)** | **0.325 (7.5e-05)** | **0.412 (3.2e-07)** | **0.297 (3.2e-04)** |
| gse21653 | 266 | **0.297 (8.1e-07)** | **0.34 (1.3e-08)** | **0.153 (0.01)** | **0.23 (1.6e-04)** | **0.316 (1.5e-07)** | **0.197 (1.2e-03)** | **0.213 (4.6e-04)** | **0.319 (1.1e-07)** | **0.211 (5.4e-04)** |
| gse25066 | 508 | **0.182 (3.5e-05)** | ND | ND | 0.094 | ND | ND | **0.115 (9.4e-03)** | ND | ND |
| gse2603 | 96 | 0.199 | ND | ND | 0.158 | ND | ND | **0.217 (0.03)** | ND | ND |
| gse28844 | 61 | 0.023 | 0.001 | 0.097 | 0.004 | -0.024 | 0.078 | 0.001 | -0.028 | 0.059 |
| gse29271 | 210 | **0.234 (6.2e-04)** | **0.325 (1.5e-06)** | **0.195 (4.7e-03)** | **0.24 (4.6e-04)** | **0.33 (9.6e-07)** | **0.196 (4.4e-03)** | **0.262 (1.2e-04)** | **0.341 (4.0e-07)** | **0.214 (1.9e-03)** |
| gse29431 | 66 | 0.12 | 0.217 | -0.032 | -0.021 | 0.225 | 0.134 | -0.132 | 0.114 | **0.27 (0.03)** |
| gse31912 | 89 | 0.12 | 0.101 | **-0.289 (6.0e-03)** | -0.03 | -0.034 | **-0.249 (0.02)** | -0.039 | 0.007 | **"-0.226 (0.03)** |
| gse3494 | 251 | **0.149 (0.02)** | ND | ND | **0.133 (0.03)** | ND | ND | **0.149 (0.02)** | ND | ND |
| gse36771 | 107 | **0.273 (4.5e-03)** | **0.343 (3.0e-04)** | **0.376 (6.5e-05)** | 0.168 | **0.27 (4.8e-03)** | **0.318 (8.6e-04)** | **0.256 (7.8e-03)** | **0.302 (1.6e-03)** | **0.381 (5.1e-05)** |
| gse42568 | 121 | **0.465 (7.5e-08)** | **0.297 (9.3e-04)** | **0.309 (5.7e-04)** | **0.404 (4.4e-06)** | **0.191 (0.04)** | **0.272 (2.6e-03)** | **0.469 (5.9e-08)** | **0.302 (7.8e-04)** | **0.337 (1.6e-04)** |
| gse46106 | 44 | 0.242 | 0.29 | 0.078 | 0.183 | 0.238 | 0.095 | 0.049 | 0.147 | 0.056 |
| gse50705 | 351 | **0.176 (9.1e-04)** | **0.341 (5.1e-11)** | **- 0.342(4.6e-11)** | **0.139 (8.9e-03)** | **0.331 (2.1e-10)** | **-0.34 (6.0e-11)** | **0.136 (0.01)** | **0.334 (1.4e-10)** | **-0.346 (2.6e-11)** |
| gse50948 | 156 | 0.008 | -0.027 | 0.07 | 0.079 | 0.083 | 0.095 | 0.067 | **0.202 (0.01)** | 0.071 |
| gse5460 | 123 | 0.168 | 0.144 | 0.131 | 0.165 | 0.159 | 0.164 | 0.14 | 0.115 | 0.123 |
| gse5462 | 116 | **0.233 (0.01)** | ND | ND | 0.124 | ND | ND | **0.229 (0.01)** | ND | ND |
| gse6885 | 21 | 0.133 | 0.23 | -0.103 | 0.125 | 0.228 | -0.138 | 0.114 | 0.211 | -0.107 |
| gse69031 | 124 | **0.246 (6.0e-03)** | ND | ND | **0.205 (0.02)** | ND | ND | **0.241 (6.9e-03)** | ND | ND |
| gse7390 | 198 | 0.083 | ND | ND | 0.101 | ND | ND | 0.117 | ND | ND |
| gse76124 | 198 | **0.423 (5.2e-10)** | **0.403 (3.8e-09)** | **0.296 (2.3e-05)** | **0.346 (5.8e-07)** | **0.359 (2.1e-07)** | **0.244 (5.3e-04)** | **0.361 (1.8e-07)** | **0.365 (1.3e-07)** | **0.239 (6.9e-04)** |
| gse8597 | 16 | 0.481 | 0.434 | 0.137 | 0.397 | 0.211 | 0.127 | 0.227 | 0.291 | 0.162 |
| gse9195 | 77 | 0.055 | 0.003 | 0.021 | 0.019 | -0.011 | 0.019 | 0.06 | 0.001 | 0.008 |

Pearson correlation between three DDR1 probes and three INSR probes calculated from Affymetrix datasets retrieved from GEO database. Bold typed number are statistically significant Person values and for the last ones p-value is reported between brackets. ND: no data reported because some probes were lacking in some datasets. GEO ID: ID of Affymetrix experiment from GEO database; Sample number: number of breast samples (tumoral and not tumoral) profiled in the specific experiment; C1-C9: different combinations of three DDR1 probes (1007_s_at, 210749_x_at, 207169_x_at) and three INSR probes (213792_s_at, 226450_at, 226216_at) for calculation of nine different Pearson values.
